# Big Tau: What We Know, and We Need to Know

**DOI:** 10.1523/ENEURO.0052-23.2023

**Published:** 2023-05-09

**Authors:** Itzhak Fischer

**Affiliations:** Department of Neurobiology and Anatomy, Drexel University College of Medicine, Philadelphia, Pennsylvania 19129

**Keywords:** cytoskeleton, development, microtubules, PNS, regeneration, tauopathies

## Abstract

Tau is a microtubule-associated protein (MAP) that has multiple isoforms generated by alternative splicing of the MAPT gene at a range of 45–60 kDa [low-molecular-weight (LMW) tau] as well as a unique isoform termed Big tau containing an additional exon 4a encoding a large projecting domain of ∼250 aa to form a protein of 110 kDa. Big tau is expressed in adult PNS neurons such as DRG neurons and specific regions of CNS such as the cerebellum in a developmental transition from LMW tau to Big tau during the postnatal period. Despite a conserved size of the 4a exons across the vertebrate phylogeny, there is no sequence homology among different species outside the Mammalia class, which underscores the focus on structural preservation of Big tau. Despite the original discovery of Big tau in the early 1990s, there has been little progress elucidating its physiological properties and pathologic implications. We propose that Big tau may be able to improve axonal transport in projecting axons and speculate on the potential protective properties in preventing tau aggregation in pathologic conditions. This perspective highlights the importance and benefits of understanding of the role of Big tau in neuronal health and disease.

## Visual Abstract

**Figure:**
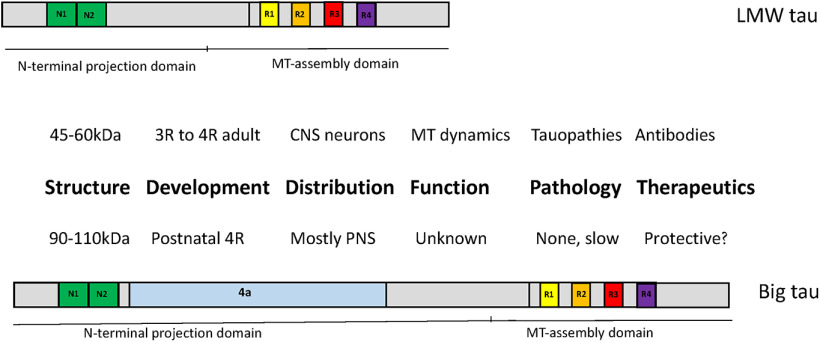

## Significance Statement

Tau has been a focus of research with respect to modulating microtubule dynamics and axonal transport, and its role in tauopathies. Big tau, which contains the large 4a exon (250aa), is expressed in PNS neurons and specific regions of the CNS neurons, increasing the size and 3D structure of tau. There has been little progress since its original discovery 30 years ago, leaving a significant gap of knowledge on how the switch to Big tau affects the properties of neurons in the context of development, disease, or injury. Here we summarize what we know about Big tau, emphasizing the scientific and clinical importance of continuing research into the function of Big tau and setting a plan for future studies.

## Introduction

Over the last 50 years, the growing interest in tau proteins can be illustrated by a simple PubMed search that identifies 5000 papers in combination with microtubules (MTs), and 18,000 in combination with tauopathies. This interest has developed in the context of the ability of tau to modulate the dynamics of MTs, axonal transport, and other contributions to neuronal cell structure, and to function as well as the pathology of neuronal degeneration in various tauopathies (Wang and Mandelkow, 2016). One of the pivotal early steps was its cloning and sequencing in 1988 (Goedert et al., 1988; Lee et al., 1988) revealing the exonal structure as the basis for generating different isoforms by alternative splicing at apparent molecular weights of 45–60 kDa (Corsi et al., 2022). It also revealed the basic domains of tau such as the N-terminal projection region, the proline-rich region, the microtubule binding region, and the C-terminal region, which have different physicochemical and functional properties (Brandt et al., 2020). In contrast, when searching PubMed for Big tau, which was cloned and sequenced in 1992 (Couchie et al., 1992; Goedert et al., 1992), revealing the addition of exon 4a to generate an isoform with an apparent molecular weight of 110 kDa, only a handful of articles (<10) can be found. Specifically, there were a few articles from the early 1990s, following the cloning of Big tau, mostly describing its unique distribution in PNS and selective regions of the CNS (Georgieff et al., 1993; Boyne et al., 1995). There is then a gap of almost 25 years in which there was just a trickle of data on Big tau, but those data were not necessarily focused on its function in health or sickness (Mercken et al., 1995; Black et al., 1996).

This apparent lack of interest is quite puzzling as Big tau has intriguing properties ranging from dramatically increasing the size of tau and likely the folding of the protein, representing the primary tau isoform expressed in adult PNS neurons, switching from low-molecular-weight (LMW) tau to Big tau in postnatal neurons, and present in selective areas of the CNS such as the visual system and cerebellum (Table 1). Thus, many years after the discovery, sequencing, and describing of its distribution, there has been no attempt to examine the functional significance of Big tau. These are low-hanging fruit that could be initially achieved using neuronal cultures and eventually *in vivo*. At our institution, when we presented this challenge to our second-year graduate students for their preliminary examination, we received straightforward proposals that included switching tau in hippocampal cultures to Big tau and switching Big tau to LMW tau in DRG cultures to examine their interactions with MTs, their effects on axonal transport, and their response to the seeding of tau aggregates. So why has the research on Big tau in the professional community remained in almost total silence? A major factor may be the strong focus on conventional tau by cell biologists studying the neuronal cytoskeleton and by neuroscientists studying neurodegeneration following the consensus targets particularly after discovering direct causation of tauopathies with the tau gene (MAPT). My hope is that, like neurogenesis, which was relegated to the backstage for a long time, Big tau is now ready for its own show.

**Table 1:** Comparing LMW tau to Big tau

	LMW tau	Big tau
Exon structure	No 4a	4a (250 aa), 4a-L (355 aa)
Alternative splicing	2, 3, 10	2, 3 (10?)
Protein size (kDa)	45–60	90–110
Developmental expression	Embryonic to adult Change from 3R to 4R	Postnatal/adult 4R
Distribution	Most of CNS neurons Non-neuronal cells	PNS neurons Selective CNS regions
Function	MT dynamics, Axonal transport Plasticity	Mostly unknown Stability?
Pathology	Tauopathies	None or slow
Therapeutics	Antibodies, knockdown	Protective?

3R, 4R, MTBD exon 10.

## What We Know (Structure and Distribution)

1. Big tau is not expressed early in development. For example, DRG and SCG neurons express LMW tau early in development and then gradually switch to Big tau postnatally with a period during which both types of tau isoform are expressed (Boyne et al., 1995; Fischer and Baas, 2020). Interestingly, following sciatic nerve crush there is a reduction in the levels of Big tau without the re-expression of the LMW isoforms (Oblinger et al., 1991). The results are consistent with a role for Big tau in adult PNS axons (e.g., MT stability, improved axonal transport) but not regrowth or regeneration. On the contrary, reducing the levels of Big tau may promote plasticity.
2. In some cases, only specific subpopulations of neurons express Big tau. For example, in adult SCGs, all neurons express Big tau (Jin et al., 2023); in DRGs, a subset expresses Big tau without LMW tau (Boyne et al., 1995); and in RGC, it appears that subpopulations of neurons express Big tau while others express LMW tau (Fischer and Baas, 2020). The uniform expression of Big tau in SCG neurons and the selective distribution of Big tau in DRG neurons in subset of small- and medium-sized neurons that express calcitonin gene-related peptide and substance P have been well documented (Boyne et al., 1995; Jin et al., 2023). The data on selective distribution of Big tau in RGCs is still lacking relative to the impressive progress in defining the heterogeneous properties of RGCs at the molecular level (Tran et al., 2019).
3. It appears that Big tau includes exons 2/3, 6, and 10 [4 MT binding domains (MTBDs)]. However, the apparent molecular size of RGCs is 90 kDa, which can be explained by skipping of exon 2/3 (my unpublished observations).

Here we have only preliminary data that set RGCs apart with a form of Big tau that lacks much of the N-terminal domain that defines the projecting domain and is known for potential interactions with plasma membrane proteins. From both Western blot and PCR analysis, it appears that the RGCs lack exons 2/3, which accounts for the lower molecular weight relative to that in PNS neurons. Analysis of the axonal transport of tau in mouse optic nerve revealed the presence of both the LMW and the 90 kDa isoforms of Big tau moving coordinately in similar rates but not cotransported with tubulin or MAP1A (Mercken et al., 1995). However, the deletion of tau did not affect axonal transport (Yuan et al., 2008) and was not correlated with the potential for regeneration or survival (Rodriguez et al., 2020), raising fundamental questions about the function of tau and Big tau in the visual system and elsewhere (discussed later).

1. The 4a exon that defines Big tau has a conserved size of ∼250 aa, decreasing to no homology along the vertebrate phylogenic tree. Thus, apes share sequence identity of 98–99% with humans, primates share 80–94% (excluding lemurs), and mammals share 40–70%; there is no homology with birds, reptiles, amphibians, or fish (Fischer, 2022). In contrast, the C-terminal domain (exons 9–13), which contains the MTBD, shows very high sequence identity of 94–98% in mammals (including the marsupial opossum), 91–93% in birds and reptiles, and 80–81% in amphibians; even in fish sequence identity remains at 50–53%. Interestingly, the N-terminal domain (exons 1–4), which contains important functional domains (Brandt et al., 2020), has also retained a higher sequence homology than the 4a exon in the range of 83–87% in mammals (with the exception of the marsupial opossum), 60–62% in birds and reptiles, and 32–28% in reptiles, diminishing only in fish, at the beginning of the vertebrate evolution of the MAPT/MAP4/MAP2 family.

What is remarkable here is the discrepancy between the low homology of 4a and its consistent size. For example, in amphibians (frog and toad), where there is no homology of the 4a exon with humans (background sequence identity, 15% and 16%), the sizes of the 4a exons are 226 and 262 aa relative to 251 aa in humans.

We have therefore speculated that the 4a exon evolved independently in different species, possibly by the process of exonization (Fischer, 2022). For example, intronic sequences or transposable elements with diverse sequences in different species could evolve into translatable exons (Sorek et al., 2004) and are expressed into proteins by alternative splicing with only the constraint of size to generate Big tau variants. The alternative model is a prototype of 4a whose sequence continuously changed along vertebrate evolution. Such an evolutionary perspective emphasizes the importance of Big tau to the vertebrate nervous system. It demonstrates that the basic structure of the MTBD present in invertebrates evolved N-terminal sequences as well as the 4a exon as a response to the growing functional and structural complexities of the nervous system. There may be some functional redundancy of LMW tau and Big tau with the isoforms of MAP2 (a/b/c/d), noting that MAPT/MAP4/MAP2 all belong to the same family (Dehmelt and Halpain, 2005).

1. The pattern of tau expression with respect to the ratio of LMW tau to Big Tau appears to be modulated by nucleoprotein granules. Experiments using neuronal cell cultures showed that formation of granules increased the ratio of Big tau and induced neuronal sprouting (Moschner et al., 2014). It is also possible that microRNAs that bind to the 4a exon of Big tau or specific exon junctions of LMW tau can also affect the pattern of tau expression. These modes of control represent transcriptional and translational mechanisms relevant to the expression of Big tau, as well as the response to stress and neurodegeneration (Cruz et al., 2019).
2. Big tau is also present in spinal motor neurons and axons in the ventral white matter as well as most cranial nerve motor nuclei such as oculomotor and trochlear (Boyne et al., 1995). Functional data in this area are lacking.
3. Another form of Big tau has been recently identified where the 4a exon is 355 aa (4a-L), which is present in some mammals and also is associated with tumorigenic cells and response to chemotherapeutic agents (Souter and Lee, 2010). Again, data in this area are quite limited.

## What We Need to Know (Function in Health and Disease)

What emerges from the limited studies so far is a tight control over the expression of Big tau, not only in selected regions of the nervous system, but sometimes selective neuronal population within the region and expression of selective variants of Big tau. This suggests the tuning of Big tau to specific functional needs, keeping a canonical range of the 4a at ∼250 aa with one exception so far of 4a-L at 355 aa, using alternative splicing of exons 2/3 but likely no phosphorylation variations, as the 4a exons seem to be almost entirely devoid of such sites. The long projection domain of Big tau may, however, limit access of kinases and phosphatases to the many phosphorylation sites of the other domains affecting both physiological properties (Kanaan et al., 2012) and hyperphosphorylation associated with aberrant tau (Wang and Mandelkow, 2016). One potential function that seems consistent with these properties is the ability of Big tau to modulate axonal transport by providing increased spacing between microtubules and decreasing axoplasmic resistance to facilitate the movement of motor proteins. In this case, we need to know whether peripheral axon hillocks, which have tightly packed MTs, exclude big tau while the region beyond the hillock has big tau. Long-projecting PNS axons with large caliber will certainly benefit from such enhancements as well as from the increase in stability (Fischer and Baas, 2020). Another vexing issue is the interpretation of experiments that analyzed the consequences of tau knockout (KO) in transgenic animals, or acutely by using viral vectors (Ke et al., 2012). It will be important to compare the loss-of-function as well as the gain-of-function differences between Big tau and LMW tau in KO animals and the types of MAPs that can compensate for their loss, respectively.

The other important aspect of Big tau function is related to the pathology of tauopathies (Chang et al., 2021; Song et al., 2021). It is reasonable to assume even without direct evidence that the major increase of tau size will affect its structural properties and possibly its propensity to aggregate. This may be analogous to MAP2a/b, a member of the MAPT family with a similar MT binding domain and a long projection domain that does not form aggregates and is not involved in the formation of neurofibrillary tangles (Xie et al., 2014). If this is the case, then PNS neurons, which are under great metabolic stress of maintaining an enormous axonal structure, may need extra protection from the liabilities of conventional tau. It could even be argued that Big tau is the original default structure providing exceptional stability that lost the 4a exons to address the need for the plasticity of CNS neurons. The undesired consequences of neurodegeneration may not have had an evolutionary pressure because they manifest late in life, without affecting reproductive advantages. What supports such theory is the observation that the PNS in general and CNS areas that express Big tau, such as the cerebellum, are less vulnerable to tauopathy-related degeneration. It would be interesting to develop 3D models comparing LMW tau with Big tau and design experiments to directly test the role of Big tau in protecting against aggregation and propagation.

What are then the therapeutic implications of the potential protective properties of Big tau? This is a tough call because it is unlikely that a therapy will use gene therapy to substitute LMW tau with Big tau in humans especially when there are no reliable biomarker indicating the potential for tauopathies for preventive treatment (unless the CRISPER-Cas technology becomes very accurate and efficient). However, an alternative splicing of 4a could be induced by factors identified in the analysis of Big tau expression. It may also be possible to deliver Big tau to reduce the degenerative process and the proliferation of aggregates.

## Synthesis

Reviewing Editor: Jeffery Twiss, University of South Carolina Decisions are customarily a result of the Reviewing Editor and the peer reviewers coming together and discussing their recommendations until a consensus is reached. When revisions are invited, a fact-based synthesis statement explaining their decision and outlining what is needed to prepare a revision will be listed below. The following reviewer(s) agreed to reveal their identity: Kenneth Kosik, Roland Brandt.

eN-OPN-0052-23

The microtubule-associated protein tau has attracted attention for its involvement in a class of neurodegenerative diseases called tauopathies. Tauopathies are characterized by post-translational changes in tau protein, most notably increased phosphorylation at selected sites, termed hyperphosphorylation. A second abnormality is the formation of aggregates that accumulate in the cell body. An important feature of tau is the presence of a complex splicing pattern that leads to the generation of developmentally and spatially regulated isoforms. Overall, this results in a complex proteoform of the protein where alterations in isoform expression can cause neurodegenerative diseases. Also, an interesting feature is the generation of even longer isoforms in some neurons, including PNS neurons such as DRG neurons. These additional isoforms have been termed “big tau”, but little is known about the functional role of these isoforms. Big Tau is clearly underexplored; thus, the reviewers and reviewing editor note that this opinion manuscript is a timely undertaking. While the authors articulate important points, the manuscript remains somewhat superficial and there are some points need clarification while others important points are omitted:

1. It has been known since the work of the Hirokawa lab in the 1990s (Harada et al. Nature 369:488-491 (1994) and many others thereafter) that tau knockout has little consequence, including in terms of the PNS. The authors should speculate on how can this be interpreted in terms of a function of Big Tau?

2. In the “what we need to know” section, the argument is put forth that Big tau localization is related to axonal spacing. Therefore, we need to know if peripheral axon hillocks which have tightly packed MTs exclude big tau while the region beyond the hillock has big tau.

3. For sentence, “The lower molecular weight of Big tau in the optic nerve relative to PNS axons may imply a correlation of the size of the protein with the length and caliber of the axons?” - This seems like a sweeping conclusion even stated with the qualification “imply.” There are many sites in the PNS where this comment could be substantiated or refuted. The optic nerve alone is not validation.

Also for the sentence “It remains however unknown why the visual system expressing Big tau while almost all other CNS neurons express only LMW tau and what function does Big tau serve in RGCs and the optic nerve?” This is a cumbersome sentence and “why question” in science is not answerable.

Finally, the sentence “I hope that the visual system experts will find the answers soon” is a rather gratuitous comment and not needed.

4. Tau has several functional domains such as the N-terminal projection region, the proline-rich region, the microtubule binding region and the extreme C-terminus. These regions have rather different physicochemical properties, for example in terms of estimated charge, degree of disorder, and isoelectric point, all of which provide some clues to possible functions (see, for example, Brandt et al. Frontiers Neurol. 11:590059(2020)). Additional information on the physicochemical properties of exon 4a would be extremely useful for the reader.

5. The authors’ Point #4 is would be greatly enhanced with a figure that shows the species and the percent identities. The huge phylogenic distances mentioned are too broad and does not support the statement “... evolved independently in different species ...” because the analysis is not broken down by species. The authors need to define exonization and what data support this evolution concept. Does conservation of the length of 4a support this hypothesis? If so, how?

Homologies should be shown more quantitatively and compared to neighboring exons that are also outside the microtubule binding domain. For example, percent identity for exons 1-4 are lumped together.

6. Tau is known to have a fairly dynamic interaction with microtubules, which has been termed kiss and hop, while also acting as a potential spacer to keep microtubules apart. Could the inclusion of exon 4a change its dynamics and spacer properties (e.g. by keeping microtubules better apart for more efficient long-distance transport by motor proteins)?

7. The authors relate Big tau to their absence in tau aggregates. They might attempt to relate this observation to the extended projection in analogy to MAP2 which also has an MT binding domain similar to tau but has a very much longer projection domain and fails to form aggregates.

8. An important aspect of tauopathies is the increased phosphorylation of tau. Most disease-associated phosphorylation events occur in the proline-rich region (172-243) and the CTR (369-441). What is known about possible phosphorylation sites in exon 4a? For example, are there potential sites for GSK3beta or other kinases that are discussed to be involved in tau hyperphosphorylation?

9. There is an interesting cell line study showing that stress granule induction changes the isoform pattern of tau from LMW tau to Big Tau, which is associated with increased process growth (Moschner et al. JBC 289:16814-16825 (2014)). Could reprogramming isoform expression play a role in the stress response to promote recovery?

## Author Response

Synthesis Statement for Author (Required):

The microtubule-associated protein tau has attracted attention for its involvement in a class of neurodegenerative diseases called tauopathies. Tauopathies are characterized by post-translational changes in tau protein, most notably increased phosphorylation at selected sites, termed hyperphosphorylation. A second abnormality is the formation of aggregates that accumulate in the cell body. An important feature of tau is the presence of a complex splicing pattern that leads to the generation of developmentally and spatially regulated isoforms.

Overall, this results in a complex proteoform of the protein where alterations in isoform expression can cause neurodegenerative diseases. Also, an interesting feature is the generation of even longer isoforms in some neurons, including PNS neurons such as DRG neurons. These additional isoforms have been termed “big tau”, but little is known about the functional role of these isoforms. Big Tau is clearly underexplored; thus, the reviewers and reviewing editor note that this opinion manuscript is a timely undertaking. While the authors articulate important points, the manuscript remains somewhat superficial and there are some points need clarification while others important points are omitted:

1. It has been known since the work of the Hirokawa lab in the 1990s (Harada et al. Nature 369:488-491 (1994) and many others thereafter) that tau knockout has little consequence, including in terms of the PNS. The authors should speculate on how can this be interpreted in terms of a function of Big Tau?

This is a complex issue involving knockout animals and acute knockout using viral vectors with some behavioral phenotypes. I added a review reference and some of my views with regard to Big tau.

2. In the “what we need to know” section, the argument is put forth that Big tau localization is related to axonal spacing. Therefore, we need to know if peripheral axon hillocks which have tightly packed MTs exclude big tau while the region beyond the hillock has big tau. Added accordingly.

3. For sentence, “The lower molecular weight of Big tau in the optic nerve relative to PNS axons may imply a correlation of the size of the protein with the length and caliber of the axons?” - This seems like a sweeping conclusion even stated with the qualification “imply.” There are many sites in the PNS where this comment could be substantiated or refuted. The optic nerve alone is not validation. Also for the sentence “It remains however unknown why the visual system expressing Big tau while almost all other CNS neurons express only LMW tau and what function does Big tau serve in RGCs and the optic nerve?” This is a cumbersome sentence and “why question” in science is not answerable.

Finally, the sentence “I hope that the visual system experts will find the answers soon” is a rather gratuitous comment and not needed. I revised accordingly. 2

4. Tau has several functional domains such as the N-terminal projection region, the proline-rich region, the microtubule binding region and the extreme C-terminus. These regions have rather different physicochemical properties, for example in terms of estimated charge, degree of disorder, and isoelectric point, all of which provide some clues to possible functions (see, for example, Brandt et al. (Brandt et al., 2020)(2020)). Additional information on the physicochemical properties of exon 4a would be extremely useful for the reader. I added more information on the different regions of tau and how they may relate to the properties of Big tau.

5. The authors’ Point #4 is would be greatly enhanced with a figure that shows the species and the percent identities. The huge phylogenic distances mentioned are too broad and does not support the statement “... evolved independently in different species ...” because the analysis is not broken down by species. The authors need to define exonization and what data support this evolution concept. Does conservation of the length of 4a support this hypothesis? If so, how? Homologies should be shown more quantitatively and compared to neighboring exons that are also outside the microtubule binding domain. For example, percent identity for exons 1-4 are lumped together. I added more details about the homology of 4a relative to the C-terminal and N-terminal. Since these data are quoted from a publishes paper, we do not have more refined analysis of individual exons in this Opinion manuscript. Same applies to the “speculation” about exonization of the 4a, which refers to the same manuscript. We added however, a note of caution (also in the original paper) that the alternative model is a prototype of 4a whose sequence continuously changed along vertebrate evolution.

6. Tau is known to have a fairly dynamic interaction with microtubules, which has been termed kiss and hop, while also acting as a potential spacer to keep microtubules apart. Could the inclusion of exon 4a change its dynamics and spacer properties (e.g. by keeping microtubules better apart for more efficient long-distance transport by motor proteins)? I agree and included this function for the role of Big tau with emphasis on projecting neurons requiring long distance transport.

7. The authors relate Big tau to their absence in tau aggregates. They might attempt to relate this observation to the extended projection in analogy to MAP2 which also has an MT binding domain similar to tau but has a very much longer projection domain and fails to form aggregates. Good point, thanks

8. An important aspect of tauopathies is the increased phosphorylation of tau. Most disease-associated phosphorylation events occur in the proline-rich region (172-243) and the CTR (369- 441). What is known about possible phosphorylation sites in exon 4a? For example, are there potential sites for GSK3beta or other kinases that are discussed to be involved in tau hyperphosphorylation? 3 I mention the lack of phosphorylation sites on the 4a and the potential effect of the long projection arm on tau phosphorylation.

9. There is an interesting cell line study showing that stress granule induction changes the isoform pattern of tau from LMW tau to Big Tau, which is associated with increased process growth (Moschner et al. JBC 289:16814-16825 (2014)). Could reprogramming isoform expression play a role in the stress response to promote recovery? Thanks, I missed this one and now added as a specific section 5
